# ﻿The curse of the uncultured fungus

**DOI:** 10.3897/mycokeys.86.76053

**Published:** 2022-02-02

**Authors:** Kessy Abarenkov, Erik Kristiansson, Martin Ryberg, Sandra Nogal-Prata, Daniela Gómez-Martínez, Katrin Stüer-Patowsky, Tobias Jansson, Sergei Põlme, Masoomeh Ghobad-Nejhad, Natàlia Corcoll, Ruud Scharn, Marisol Sánchez-García, Maryia Khomich, Christian Wurzbacher, R. Henrik Nilsson

**Affiliations:** 1 Natural History Museum, University of Tartu, Vanemuise 46, Tartu 51014, Estonia; 2 Department of Mathematical Sciences, Chalmers University of Technology and University of Gothenburg, Göteborg, Sweden; 3 Department of Organismal Biology, Uppsala University, Uppsala, Sweden; 4 Department of Mycology, Real Jardín Botánico-CSIC, Madrid, Spain; 5 University of Gothenburg, Department of Biological and Environmental Sciences, Box 461, 405 30 Göteborg, Sweden; 6 Technical University of Munich, Chair of Urban Water Systems Engineering, Am Coulombwall 3, 85748 Garching, Germany; 7 Iranian Research Organization for Science and Technology, Department of Biotechnology, PO Box 3353-5111, Tehran 3353136846, Iran; 8 University of Gothenburg, Gothenburg Global Biodiversity Centre, Department of Biological and Environmental Sciences, Box 461, 405 30 Göteborg, Sweden; 9 University of Gothenburg, Department of Earth Sciences, Box 460, 405 30 Göteborg, Sweden; 10 Swedish University of Agricultural Sciences, Department of Forest Mycology and Plant Pathology, Uppsala, Sweden; 11 University of Bergen, Department of Clinical Science, Box 7804, 5020 Bergen, Norway

**Keywords:** Data interoperability, data mining, DNA barcoding, scientific practice, species identification, taxonomic annotation

## Abstract

The international DNA sequence databases abound in fungal sequences not annotated beyond the kingdom level, typically bearing names such as “uncultured fungus”. These sequences beget low-resolution mycological results and invite further deposition of similarly poorly annotated entries. What do these sequences represent? This study uses a 767,918-sequence corpus of public full-length fungal ITS sequences to estimate what proportion of the 95,055 “uncultured fungus” sequences that represent truly unidentifiable fungal taxa – and what proportion of them that would have been straightforward to annotate to some more meaningful taxonomic level at the time of sequence deposition. Our results suggest that more than 70% of these sequences would have been trivial to identify to at least the order/family level at the time of sequence deposition, hinting that factors other than poor availability of relevant reference sequences explain the low-resolution names. We speculate that researchers’ perceived lack of time and lack of insight into the ramifications of this problem are the main explanations for the low-resolution names. We were surprised to find that more than a fifth of these sequences seem to have been deposited by mycologists rather than researchers unfamiliar with the consequences of poorly annotated fungal sequences in molecular repositories. The proportion of these needlessly poorly annotated sequences does not decline over time, suggesting that this problem must not be left unchecked.

## ﻿Introduction

DNA sequencing enables researchers to explore environmental habitats such as soil, wood and water for fungal diversity. A common choice of genetic marker for such pursuits is the nuclear ribosomal internal transcribed spacer (ITS) region, the formal fungal barcode (Schoch et al. 2012). Assessment of the taxonomic affiliation of newly-generated ITS sequences is typically accomplished through similarity-based searches in databases, such as the International Nucleotide Sequence Database Collaboration (INSDC; Arita et al. 2021) and UNITE (Nilsson et al. 2019). A number of factors combine to impede such assessments. For instance, sequences may be subject to distortive technical complications, such as low read quality or chimeric unions (Zinger et al. 2019). Interpretation of match statistics across a genetic marker that features both very conserved and very variable parts – such as the ITS region – can furthermore present a challenge and it seems unlikely to come up with well-defined similarity thresholds to demarcate the species and other ranks in a unified way across the entire fungal kingdom (Abarenkov et al. 2016). However, the foremost challenge is probably of taxonomic nature: reference ITS sequence data are available for a modest 25% of the ~150,000 formally described species of fungi, less than 2% of the estimated 2.3–6 million extant species of fungi (http://www.speciesfungorum.org; Hawksworth and Lücking 2017; Baldrian et al. 2021). Thus, the public sequence databases clearly suffer from a significant taxon sampling problem when it comes to their coverage of fungal biodiversity.

Roughly 42% (326,062) of the 767,918 full-length Sanger-derived fungal ITS sequences in the INSDC (November 2020) lack a full species name and 29% (95,055) of these are not annotated beyond the kingdom level (e.g. “uncultured fungus” from the environmental (ENV) sample division and “fungal sp.” from the plants and fungal (PLN) division; Sayers et al. 2021). Some proportion of these sequences are truly unidentifiable at present and stem from the many “dark lineages” or less well explored parts of otherwise well-studied groups of the fungal tree of life (Tedersoo et al. 2020; Lücking et al. 2021). Others, however, represent sequences for which some more meaningful taxonomic annotation would have been only a sequence similarity search in, for example, BLAST (Altschul et al. 1997) away at the time of sequence deposition. These sequences can be thought of as false negatives: it is well-known (or straightforward to find out) what taxon they represent, yet their annotation does not convey that information. This lack of meaningful taxonomic annotations hurts the study of fungi. The sheer number of false negatives in BLAST match lists introduces uncertainty in what should have been straightforward taxonomic decisions, often with the result that researchers adopt these uninformative names for their newly-generated sequences in what has been dubbed the “percolation” or “snowballing” effect (Gilks et al. 2002). This leads to mycology-orientated articles with low taxonomic resolution, something that mycology clearly could do without. In addition, many researchers are reluctant to include sequences without taxonomic annotation in their studies, thus missing out on potentially valuable information in phylogenetics, ecology, biogeography, and other aspects (Ryberg et al. 2008; Bonito et al. 2010; Nilsson et al. 2011; Fryssouli et al. 2020).

**Figure 1.**
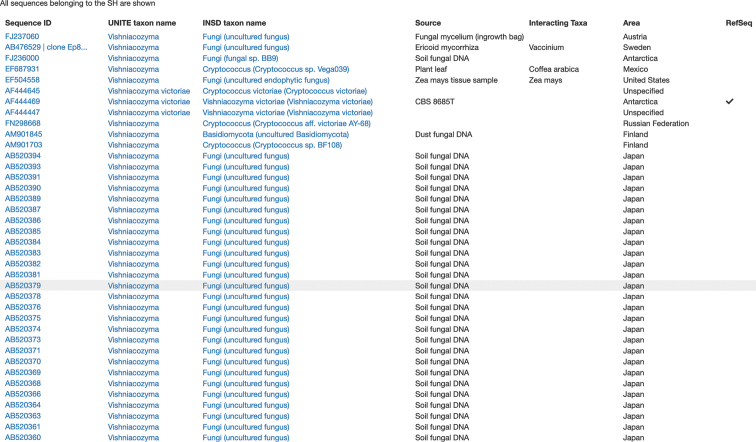
A screenshot from species hypothesis SH1159264.08FU (*Vishniacozymavictoriae*; https://dx.doi.org/10.15156/BIO/SH1159264.08FU) in UNITE. Identifying a *Vishniacozymavictoriae*ITS sequence to at least the genus level is trivial, yet the screenshot hints at the swathes of kingdom level-annotated *Vishniacozymavictoriae* sequences regularly deposited in the INSDC. SequenceID – INSDC accession number. UNITE taxon name – taxonomic annotation in UNITE. INSD taxon name – original taxonomic annotation in INSDC. RefSeq – indicates a type-derived sequence. More than thirty studies have deposited kingdom-level annotations in this species hypothesis. The ones shown primarily stem from Nishizawa et al. (2010).

Many of the present authors are curators of specific taxonomic groups in the UNITE database. In that role, we revisit our favourite fungal groups and multiple sequence alignments after each incremental update with new INSDC sequences. Unfortunately, we regularly find that previously tidy and well-annotated species hypotheses have been watered down by tens to hundreds of sequences of the “uncultured fungus” kind (Figure 1). Spending valuable curation time on handling this needless and avoidable problem is a breeding ground for frustration. Does this problem extend beyond the relatively limited number of primarily basidiomycete species hypotheses that the present authors monitor out of personal interest? If it does, mycology is at the receiving end of a seemingly never-ending stream of unnecessarily uninformative taxonomic annotations, much to its detriment. We set out to establish the background and context of the “uncultured fungus” problem through three main questions: (i) for what proportion of the 95,055 fungal ITS sequences that lack taxonomic annotation beyond the kingdom level is that lack justified due to the absence of relevant reference sequences with richer taxonomic annotations at the time of sequence deposition?; (ii) were the unjustified “uncultured fungus” sequences generated by mycologists (who perhaps should know better) or do they stem from other scientific disciplines?; and (iii) is the proportion of needlessly imprecise annotations going down over time? We pursed these questions considering all 767,918 more or less full-length, Sanger-derived fungal ITS sequences that were assigned to a UNITE species hypothesis as of November 2020. Our results suggest that “uncultured fungus” and “fungal sp.” are labels that are routinely attached to newly-generated sequences regardless of whether a more informative annotation would have been available or not. A surprisingly high proportion of these sequences stem from mycologists – researchers one would think would know that mycology does not stand to benefit from such actions.

## ﻿Materials and methods

### ﻿Defining the reference corpus

We targeted all 767,918 full-length, Sanger-derived fungal ITS sequences (annotated as such) in the INSDC (November 2020) as mirrored in the UNITE species hypotheses release 8. For each such sequence, UNITE extracts and stores relevant metadata from the GenBank flat file format (https://www.ncbi.nlm.nih.gov/Sitemap/samplerecord.html). Sequence quality control is part of the species hypotheses generation and seeks to exclude clear cases of, for example, chimeras and low read quality sequences through tools, such as USEARCH (Edgar 2010) and ITSx (Bengtsson-Palme et al. 2013). All sequences are first clustered at 80% similarity in USEARCH to obtain compound clusters. These clusters typically revolve around the family/subfamily/genus level. Each compound cluster is then clustered into species hypotheses (SHs) at distance thresholds 0.0% through to 3.0% in steps of 0.5%. These can be thought of as entities roughly at the species level.

UNITE uses the NCBI Taxonomy classification (Schoch et al. 2020) as the taxonomic backbone, supplemented with modifications from Index Fungorum (http://www.indexfungorum.org), MycoBank (Robert et al. 2013), Tedersoo et al. (2018) and the UNITE user base. UNITE uses the taxonomic annotations of the sequences in each SH to determine the taxonomic affiliation of the SH. The algorithm underpinning this determination ignores low-resolution annotations as long as they are not contradictory: an SH that contains five sequences annotated as *Amanitamuscaria* and one annotated as “Basidiomycota sp.” will be scored to represent *Amanitamuscaria*. However, a (compromised) two-sequence SH containing *Amanitamuscaria* and *Cantharelluscibarius* will be assigned to the most resolved level where their classifications are compatible, in this case the class *Agaricomycetes*. In this way, each sequence and species hypothesis in UNITE are assigned to the most resolved position possible in the fungal tree of life given the available information. For this study, we targeted all 95,055 sequences originally released in INSDC without any more refined taxonomic annotation than the kingdom level (such as “uncultured fungus” and “fungal sp.”), irrespective of whether UNITE – or a UNITE user – subsequently had been able to assign a more refined name to it.

### ﻿Mimicking BLAST searches

The fact that UNITE stores the INSDC initial release date for each sequence allowed us to build a map of what sequences were available in INSDC at any time. We wanted to capture what we feel are the two most common scenarios of INSDC sequence deposition, namely: (i) a user deposits sequences for immediate release and (ii) a user deposits sequences for release, pending acceptance of the underlying manuscript. Thus, for each sequence A that was only annotated at the kingdom level, we considered all sequences that were released at least seven days before A as being available for BLAST searches by the authors of A. This leaves room for the authors of A to have done a final double check of the taxonomic affiliation of their soon-to-be-released sequences, including A, prior to setting them free.

We sought to recreate what such a BLAST search would have looked like to the authors of A with respect to closely matching (≥ 97% similarity) sequences (the topmost, high-scoring sequences in a BLAST hit list), as well as sequences that produced reasonable (≥ 80% similarity), but not top-scoring, matches to A. This captures our experience of BLAST – most users, it seems to us, do not bother looking beyond the first ~20 BLAST matches for clues to the taxonomic affiliation of a query sequence. For this “closely matching sequences” dataset, we examined the 3.0% species hypothesis of each kingdom-level sequence for the presence of sequences at least 7 days older than the kingdom-level sequence. Any such sequences were examined for their INSDC taxonomic annotation from kingdom to the species level. This allowed us to build a view of what the author of the kingdom-level sequence would have seen, had they done a BLAST search prior to the release of the kingdom-level sequence. For the “reasonable, but not top-scoring, matches” dataset, we, instead, considered the (≥ 80% similarity) compound cluster where each kingdom-level sequence was found. This allowed us to model the scenario where the kingdom-level sequence authors progressed further down in the BLAST hit list for taxonomic clues, plus the scenario where there were no close BLAST matches to begin with.

### ﻿Metadata assessment and statistical analyses

We examined the GenBank FEATURES field for information on the country of collection of each sequence to get a feeling for whether kingdom-level sequences and the sequences annotated beyond the kingdom level stemmed from dramatically different sampling areas. Some two percent (2,049) of the sequences annotated only at the kingdom level (e.g. “uncultured fungus”) were found to initially lack an explicit country of collection, yet stem from a published or otherwise available (e.g. a pre-print) study (as opposed to being a “Direct submission” or an “Unpublished” INSDC submission). Similarly, some 7% (48,540) of the sequences with at least a phylum-level annotation (e.g. “Ascomycota sp.” and “*Rhizoplaca* sp.”) were found to lack an explicit country of collection, but to stem from a published or otherwise available study. These sequences offer some hope of restoration of the missing country of collection through recourse to the presumed underlying publication; sequences merely listed as “Direct submission” or “Unpublished” do not, in our experience (e.g. Abarenkov et al. 2016). Based on published information and online queries in, for example, preprint repositories, we thus made an effort to restore the country of collection for all 2,049 kingdom-level sequences whose GenBank REFERENCE field specified a tangible publication (published, preprint or in press). We repeated this task for a random 2,049 of the 48,540 sequences annotated to at least the phylum level. Ideally, we would have targeted all 48,540 phylum-level sequences, but this substantial task was deemed beyond the capacity of the present set of authors (cf. Durkin et al. 2020). Insofar as the underlying publications could be tracked down and the country of collection could be derived from the paper (or through contacting its authors), the country of collection was added to UNITE and used in this study.

When the GenBank REFERENCE field specified a scientific journal, we used the journal name as a proxy for whether the author(s) of each sequence were mycologists or not. We made the admittedly crude assumptions that a mycologist is someone who publishes in a mycological journal; that only mycologists publish papers in mycological journals; and to only consider the 29 journals listed under “Mycology” in Web of Science (November 2020; Suppl. material 1) as mycological journals. All other journals and sequence authors were scored as non-mycological.

The year of deposition of each sequence was assessed to examine whether the proportion of kingdom-level INSDC depositions fluctuated over time (2001–2020).

## ﻿Results

### ﻿Taxonomic resolution

**Figure 2.**
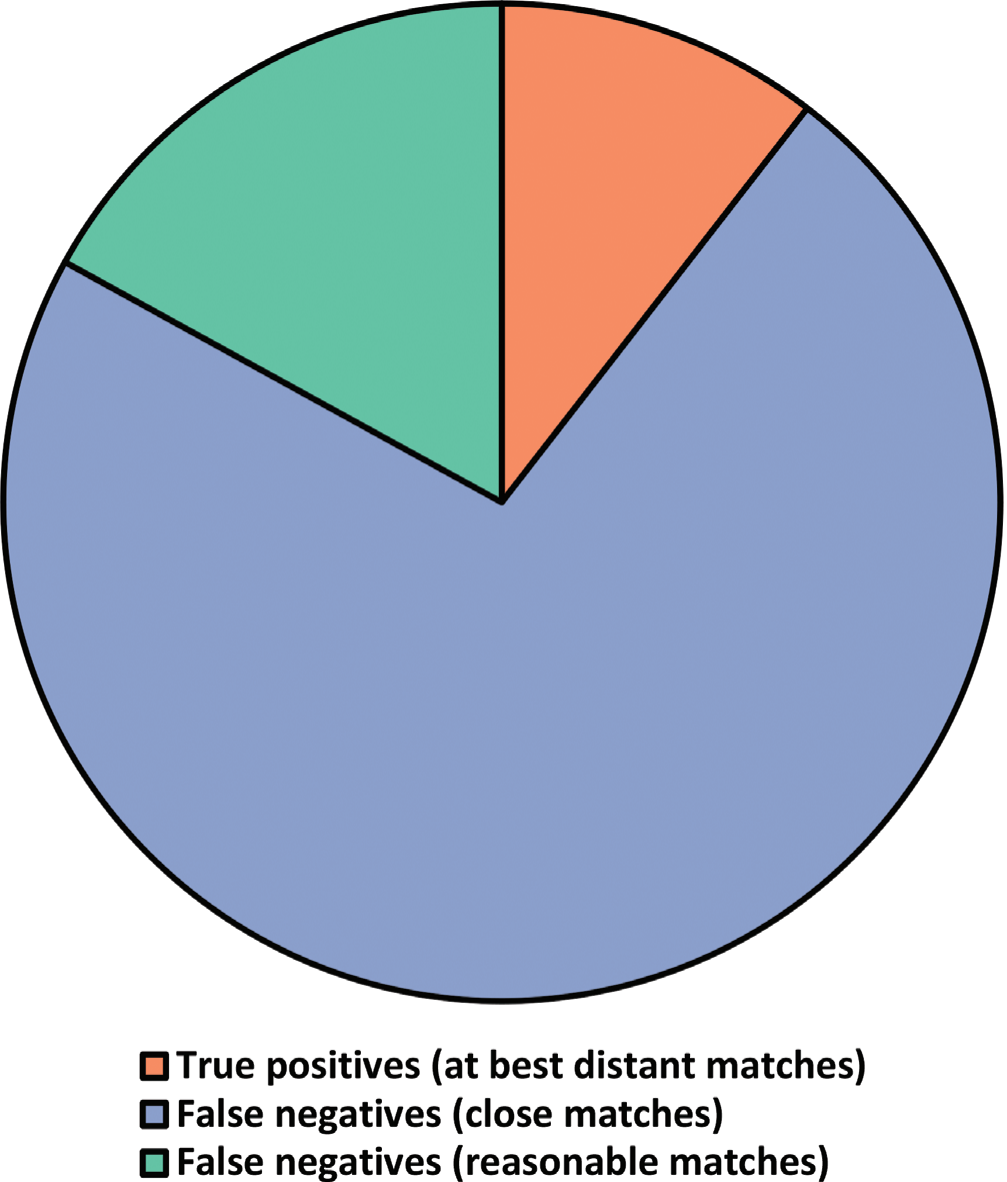
Pie chart representing all the 95,055 kingdom-level ITS sequences and the proportion of these that were true-positives (had no or only very distant taxonomically more well-annotated BLAST matches at the time of sequence deposition/release; red, 10%), false-negatives (had only reasonable matches; green, 17%) and false-negatives (had close matches; blue, 73%). The chart suggests that nearly all kingdom-level fungal ITS sequences in INSDC could have been given a more taxonomically-resolved name at the time of sequence deposition/release.

Regarding our attempt to mimic BLAST users who only consider matches with very high match scores, we found that a full 68,929 (73%) of the 95,055 sequences annotated only at the kingdom level (Fungi) were false negatives (Figure 2). A name at the class, order, family, genus, and species level was available in the corresponding UNITE species hypothesis (and would have been available amongst the top-scoring BLAST matches) for 71%, 70%, 67%, 64% and 60% of the sequences, respectively. The BLAST hit list would have contained an average of 74% sequences annotated to at least the phylum level; 69% to the class level; 67% to the order level; 62% to the family level; 54% to the genus level and 38% to the species level. The median number of sequences in a non-singleton species hypothesis was 77 at the time of deposition of the query sequence at hand.

If we include the “reasonable, but not top-scoring, matches” from the corresponding compound cluster (i.e. sequences that would have appeared further down in the BLAST hit list) in these statistics, we found that 85,093 (90%) of the 95,055 sequences annotated only at the kingdom level were false negatives (Figure 2). A name at the class, order and family level was available for 88%, 88% and 87% of the sequences, respectively. The BLAST hit list would have contained an average of 75% sequences annotated to at least the phylum level; 70% to the class level and 68% to the order level. The average non-singleton compound cluster contained 386 sequences at the time of deposition of the query sequence at hand.

### ﻿Metadata

**Figure 3.**
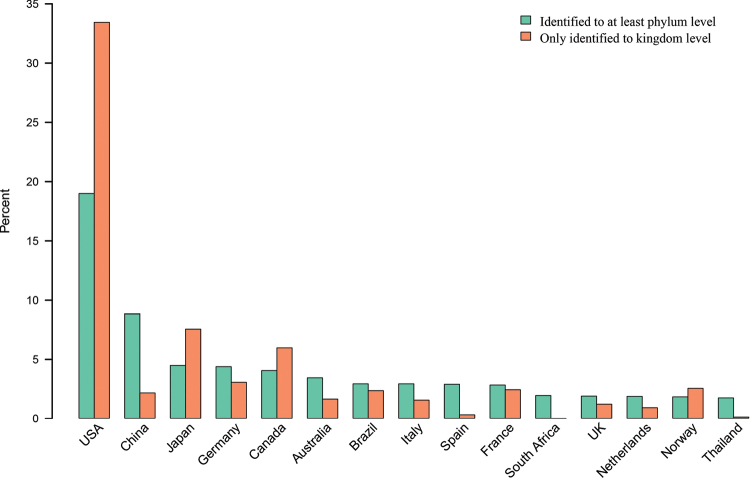
The top 15 most common countries of collection for the publication-associated sequences annotated at or beyond the phylum level (green) expressed as the proportion of the sequences stemming from each country out of all phylum-level-and-beyond sequences. The corresponding country for publication-associated sequences annotated only at the kingdom level (orange) is similarly expressed as the proportion of sequences stemming from that country out of all kingdom-level sequences. The figure is ordered in decreasing order by the country of collection for the phylum-level sequences.

Initially, 2,049 (2.2%) of the publication-associated kingdom-level sequences were found to lack information on country of collection. The corresponding number was 7% (48,540) for the publication-associated sequences with at least a phylum-level annotation. We were able to restore the country of collection for 1,983 (96.8%) of these kingdom-level sequences and 1,812 (89.3%) of these phylum-level sequences. The newly-obtained countries of collection were deposited in UNITE for each sequence to facilitate further mycological enterprises by UNITE users. Figure 3 shows the 15 most common countries of collection for the sequences annotated to at least the phylum level, overlaid with the corresponding results from the kingdom-level dataset. The two 15-country sets share 10 (67%) countries, primarily from parts of the world that are relatively well-studied from a mycological point of view. Several countries known as veritable hotspots for fungal diversity – for example, Thailand and Brazil (Hyde et al. 2018; Menolli and Sánchez-García 2020) – report dramatically lower proportions of kingdom-level sequences than do some countries with a more well-studied mycobiota. The figure suggests that factors other than lack of reference sequences are behind the prevalence of kingdom-level sequences.

**Figure 4.**
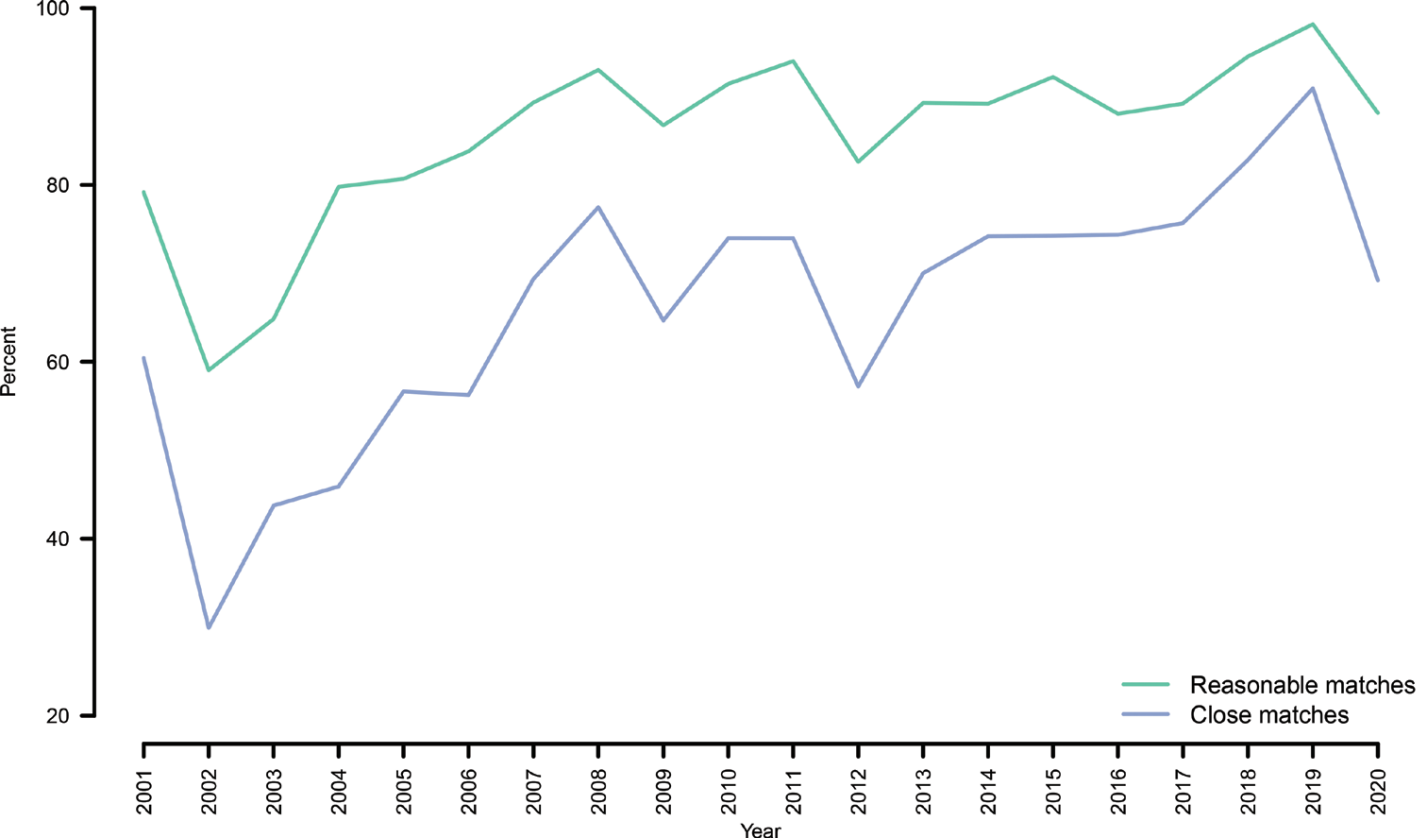
The proportion of false-negative sequences (had reasonable matches; green) and false-negative sequences (had close matches; blue) out of all kingdom-level sequences over time (2001-2020). The figure suggests that the act of taking sequence annotation very lightly is not in an abating trend. The data for 2020 extend through early November 2020 and are thus partial.

For the “closely matching sequences” scenario, we found that 22% (21,205) of the full INSDC set of kingdom-level sequences, for which a more resolved name would have been only a BLAST search away, were generated by mycologists (following our admittedly crude definition of a mycologist). When, instead, considering the fully identified sequences, 182,402 (27.1%) were deposited by mycologists. The proportion of false-negative INSDC depositions does not decline over time (Figure 4).

## ﻿Discussion

### ﻿Overall results

The present paper examines the corpus of reasonably full-length public fungal ITS sequences not annotated to any meaningful taxonomic level. We found that our initial, UNITE curation-based hunches were largely right: reasons other than lack of established taxonomy and available reference sequences lie behind the lack of resolved taxonomic annotations for the overwhelming majority of these sequences. A full 12% of the 767,918 sequences in our dataset were annotated only to kingdom level – and in at least 73% of these cases seemingly without clear justification. In fact, for 64% of these sequences, an annotation to at least the genus level seems to have been possible and only a BLAST search away at the time of sequence deposition/release. The tendency of researchers not to name fungal sequences beyond the kingdom level, even when this would have been perfectly possible, does not seem to go down over time (Figure 4). One cannot help but ponder a future scenario where BLAST searches become increasingly tedious and time-consuming to interpret – and, in fact, may not be meaningful at all in some cases. This is acting out in a time when the opposite should be the case – BLAST searches become increasingly informative and easy to interpret – owing to rapid taxonomic progress in mycology through initiatives such as Yuan et al. (2020) and Tedersoo et al. (2020).

It would somehow have been nice to conclude that mycology is the victim of the decisions of non-mycologist researchers: only non-mycologists are behind the countless “uncultured fungus” depositions. Our results are not in line with this though; mycologists seem to be behind more than one fifth of these sequences. We find this remarkable, considering that mycology is often touted as an overlooked and easily dismissed discipline (Pautasso 2013). As such, mycology should surpass, rather than dodge, expectations. It does not really seem to be happening though. We feel that mycologists are not in a robust position to accuse others of taking fungi too lightly, if mycologists themselves take fungi too lightly. The act of claiming that mycology needs more money, without backing that claim by robust and reproducible data, may well prove to be counterproductive (Durkin et al. 2020).

Our results make it painfully clear that human nature, rather than lack of taxonomic information and resolution, is the cause of the lion’s share of the kingdom-level annotations. Indeed, more than 70% of the kingdom-level sequences belong to lineages for which an established Latin name – and at least one reference sequence annotated accordingly – were readily available at the time of sequence deposition. This begs the question why those sequence authors did not go looking for that information to begin with. One can think of many answers: lack of mycological or bioinformatics expertise, lack of money/time, a research focus other than taxonomy, wanting journal policies on metadata richness and availability and, indeed, lack of a perceived good reason to take the time to do it in the first place. All those reasons can be countered one way or the other. For instance, any environmental sequencing effort likely to unravel fungi – although they may not target fungi or taxonomic aspects specifically – should always include a mycologist as well as a bioinformatician to maximise resolution in the analysis, but also the data deposition step. Grant applications should be written in such a way to provide sufficient time and resources for reproducible down-the-road data handling and not just the field and sequencing expenses. Similarly, journal policies on data availability should ideally be extended – and enforced – to also include aspects of data annotation and re-usability, perhaps to the extent that any pending INSDC entries to be released upon publication of the study must be submitted to the journal for review alongside the other manuscript files. Above all, individual research efforts should be seen not only as a way to increase the length of one’s CV and to meet promises to funding agencies, but also as a contribution to the ever-growing corpus of scientific – mycological – knowledge. In fact, we speculate that this last issue is the main reason behind the findings of the present study. Researchers do not perceive their sequence data as atomised contributions to science and, thus, fail to take the steps that would have enabled meaningful use of those sequences beyond the study at hand.

The present results dispel the assertion that only mycologists are in a position to add to our growing knowledge of the fungal kingdom. This, in turn, suggests that mycologists should make it as easy as possible for anyone to make use of, but also add to, the corpus of mycological data. After all, DNA sequences form a key component of contemporary mycology (Crous et al. 2021; Lücking et al. 2021). Thus, significant mycological expertise should not be needed to arrive at reasonable conclusions – such as a genus-level annotation – from BLAST hit lists. As mycologists, we need to take the time to annotate our sequences accordingly. Annotating newly-generated sequences to some reasonable taxonomic level – say the genus or order level in the case of environmental sequences – is, however, a process that takes time. As the list of sequences runs into the hundreds and sometimes more, we could be talking days. However, what many mycologists do not seem to realise is that there is always room for more co-authors on a scientific paper. That room is clearly not maximised right now – we found an average of 4.9 co-authors per study in the 58,898 studies behind our full 767,918-sequence dataset. The non-trivial number of kingdom-level annotations testifies to the many aspiring or junior researchers who could have received training in robust sequence annotation and then been asked to annotate the newly-generated sequences to some more meaningful level (e.g. class, order or genus) prior to deposition in exchange for a non-prominent co-authorship – but who never got the chance (Ryberg et al. 2016). We find this hard to swallow.

### ﻿Observations on sequence annotation

It is painful to come across sequences that are annotated as “uncultured fungus” or “fungal sp.” in INSDC, but that are deeply nested (and sometimes even well annotated) in well-supported clades in phylogenetic trees of, for example, *Fusarium*, Helotiales, and *Lactarius* in the associated publications. The present study argues that taxonomic annotations of the “uncultured fungus” kind should be reserved for cases where taxonomic annotation beyond the kingdom level was attempted, but came up short. Then users would know that each such sequence carries a non-trivial potential for taxonomic discovery – you could even argue that such sequences would be amongst the most interesting and exciting of all fungal sequences. Right now, however, the “uncultured fungus” label is used as a catch-all device whose routine use serves to mask the presence of truly unidentifiable fungi. Many researchers seem to shun unidentified sequences also in situations where these sequences clearly should have been considered (Nilsson et al. 2011). Improved taxonomic annotation is a way out of this dilemma.

Phylogenetic analysis is probably the most robust way to assess the taxonomic affiliation of sequences and hence to annotate sequences. However, we acknowledge that not all studies use phylogenetic approaches to begin with and that phylogenetic analysis may not be applicable in all situations. Fortunately, similarity-based searches, such as BLAST in INSDC, will take you a long way. By ticking the GenBank-BLAST box “Exclude: Uncultured/environmental sample sequences”, a more taxonomy-orientated picture is likely to emerge. We feel that a sequence that produces a long list of, say, robust *Fusarium* matches – when both BLAST coverage and similarity are considered closely (Nilsson et al. 2012) – should be annotated as “uncultured *Fusarium*” or perhaps “uncultured Nectriaceae”. Most taxonomic contradictions in BLAST hit lists can be resolved by further restricting the searches to the largely type-derived NCBI RefSeq Targeted Loci ITS Project (GenBank identifier PRJNA177353; Schoch et al. 2014). Judging by the results of the present study, these simple steps would have taken the edge off the majority of the sequences currently bearing only a kingdom-level annotation.

We would like to stress that annotating sequences is always a balance between under- and over-annotation. There is no shortage of incorrectly annotated fungal sequences in the public repositories (Hofstetter et al. 2019) and we certainly do not want this study to give rise to even more. Thus, we would be happy to see any of the names “uncultured *Fusarium*”, “uncultured Nectriaceae”, “uncultured Hypocreales”, “uncultured Sordariomycetes” and “uncultured Ascomycota”, depending on what the data at hand showed. This is one of the reasons why we feel that taxonomic expertise should be involved also in sequencing efforts that do not pursue taxonomic questions explicitly. While it borders on the impossible to algorithmise threshold values for when a sequence can be safely annotated to some specific level, rough guidelines are available. Based on the ITS2 subregion, Tedersoo et al. (2014) “typically” used the global similarity thresholds 90, 85, 80, and 75% identity for assigning operational taxonomic units to the genus, family, order, and class level, respectively. We take the caveat “typically” to refer to taxonomic expertise, because it makes little sense to insist that these threshold values will always hold true. They offer guidance, but in the face of uncertainty, we argue that it is preferable to annotate a sequence at the parental lineage, such as “uncultured Nectriaceae”, as opposed to a more tentative “uncultured *Fusarium*”.

Tedersoo et al. (2014) do not specify when a sequence should be annotated at the species level; indeed, sequences were not annotated at the species level in that study. We agree with this move and we personally do not annotate newly-generated environmental sequences to the species level other than in very rare and particularly unequivocal cases. After all, there are many examples of clearly distinct species that have identical ITS sequences (Abarenkov et al. 2016) and, in the absence of other evidence, it is simply not always possible to derive a robust species-level identification based on ITS data. At a more general level, this study advocates taxonomic annotations at the level warranted by the data as interpreted by a knowledgeable mycologist. That level is typically not that of species, but as this study shows, it is also not that of kingdom.

### ﻿Potential shortcomings of the present study

The present study should be viewed as a rough estimation of the reasons why we keep seeing INSDC submissions of the “uncultured fungus” kind. Many aspects of the present study are clearly hard to algorithmise. For instance, in our mimicking of BLAST searches, we used default BLAST settings and a single version of BLAST. However, the BLAST output may have looked somewhat different to a user with non-default parameter values or another version of BLAST. It is, furthermore, difficult to model human behaviour when it comes to processing and interpreting BLAST hit lists. One can also think of cases where the sequence authors did, in fact, do BLAST searches, but were presented with contradictory information: “Ascomycota sp.” and “Basidiomycota sp.”. In our experience, it is often easy to single out and resolve many misannotated sequences, based on the annotations of the other relevant BLAST hits – a single Lactarius (Basidiomycota) annotation in a large group of Fusarium (Ascomycota), for instance – but we can certainly see why some users would feel uncomfortable doing this. The magnitude of this problem appears limited, as 0.5% of the SHs and 1.8% of the compound clusters contained annotation conflicts at the phylum level. Complications such as these, nevertheless, suggest that our estimate that more than 70% of the kingdom-level annotations are false negatives may be off by several percentage units. That said, many of our parameter settings – such as the permissive single-linkage clustering underlying the SH generation – were deliberately set to be very forgiving. We, therefore, argue that at least the order of magnitude of our estimate is reasonable. Our estimate is, furthermore, in line with our admittedly basidiomycete-centric experience of UNITE sequence curation.

The scoring of sequence authors as mycologists or non-mycologists, based on the journal of the underlying publication, is clearly a move that will prove to be wrong in many cases. We are well aware – and welcome – that also non-mycologists publish their findings in mycological journals. Conversely, mycologists often – and rightfully – seek to publish their findings beyond mycological journals. Finally, Web of Science is not an ideal arbiter of what is mycology and what is not, given that there are many mycological journals that do not yet have a formal impact factor. Thus, while we agree that these shortcomings haunt our estimate that 22.3% of the kingdom-level sequences were submitted by mycologists, it is not immediately clear whether our estimate is biased towards, or away from, mycologists. Our estimate is clearly so high that it would be counter-intuitive to argue that only non-mycologists are behind it.

## ﻿Conclusions

The study of fungi is being reshaped by the many novel and hitherto nameless fungal lineages unearthed by environmental sequencing efforts (Lücking et al. 2021). However, the study of fungi is simultaneously being watered down by the needless yet continual deposition of sequences of low-resolution taxonomic annotations for taxa, for which a more appropriate annotation would have been only seconds away should the underlying authors have taken the time to look. The curse of the uncultured fungus is that these two cases, at least at a cursory glance, are hard to tell apart. We urge all members of the scientific – and particularly the mycological – community to reconsider their stance on batch, haphazard sequence annotation. It is a game without clear winners, but where the scientific community – and particularly mycology – certainly comes out on the losing end. This is not in anybody’s interest. Mycology is under enough strain already without having to grapple with the consequences of negligence and the urge to save time for oneself, even if at the expense of others.

## Supplementary materials

Supplementary material 1 A list of the 29 journals under the Web of Science heading “Mycology” as of November 2020 Data type Text https://binary.pensoft.net/file/641042This dataset is made available under the Open Database License (http://opendatacommons.org/licenses/odbl/1.0/). The Open Database License (ODbL) is a license agreement intended to allow users to freely share, modify, and use this Dataset while maintaining this same freedom for others, provided that the original source and author(s) are credited.Kessy Abarenkov, Erik Kristiansson, Martin Ryberg, Sandra Nogal-Prata, Daniela Gómez-Martínez, Katrin Stüer-Patowsky, Tobias Jansson, Sergei Põlme, Masoomeh Ghobad-Nejhad, Natàlia Corcoll, Ruud Scharn, Marisol Sánchez-García, Maryia Khomich, Christian Wurzbacher, R. Henrik Nilsson
