# Supplementary material for: ﻿The curse of the uncultured fungus
Source: MycoKeys. 2022 Feb 2;86:177–94. doi: 10.3897/mycokeys.86.76053 (PMC8828591; doi:10.3897/mycokeys.86.76053)
Supplement: Supplementary material 1 — A list of the 29 journals under the Web of Science heading “Mycology” as of November 2020 [file mycokeys-86-177-s001.pdf]

List of journals

Cryptogamie Mycol.
FEMS Yeast Res.
Fungal Biol.
Fungal Biol. Rev.
Fungal Divers.
Fungal Ecol.
Fungal Genet. Biol.
IMA Fungus
J. Fungi
J. Mycol. Med.
Lichenologist
Med. Mycol.
MycoKeys
Mycobiology
Mycol. Prog.
Mycologia
Mycopathologica
Mycorrhiza
Mycoscience
Mycoses
Mycosphere
Mycotaxon
Mycotoxin Res.
Persoonia
Rev. Iberoam. Micol.
Stud. Mycol.
Sydowia
World Mycotoxin J.
Yeast
